# Association between malnutrition and negative health outcomes in Italian older adults: findings from the ilSIRENTE study

**DOI:** 10.3389/fnut.2025.1585310

**Published:** 2025-06-04

**Authors:** Hélio José Coelho-Júnior, Riccardo Calvani, Andrea Russo, Francesco Landi, Emanuele Marzetti

**Affiliations:** ^1^Fondazione Policlinico Universitario Agostino Gemelli IRCCS, Rome, Italy; ^2^Department of Geriatrics, Orthopedics and Rheumatology, Università Cattolica del Sacro Cuore, Rome, Italy

**Keywords:** undernutrition, falls, fractures, disability, death

## Abstract

**Introduction:**

Malnutrition has been shown to contribute to adverse outcomes such as falls, fractures, and disability in older adults. However, findings remain conflicting, partly due to differences in operationalization methods. The recent adoption of the Global Leadership Initiative on Malnutrition (GLIM) criteria has shown promise, but evidence—especially in community-dwelling populations—is still limited and inconclusive. Therefore, the present study examined the associations between malnutrition and health-related parameters, including falls, fall-related fractures, disability, and mortality, in community-dwelling older adults.

**Methods:**

This prospective cohort study involved octogenarians who lived in the mountain community of the Sirente geographic area in Central Italy. Malnutrition was defined according to the GLIM criteria. History of falls, incident falls, fall-related fractures, and disability status according to basic activities of daily living were recorded over 2 years. Survival status was obtained from participants’ general practitioners and verified through the National Death Registry, up to 10 years from enrollment. Binary, ordinal, and Cox regression analyses were performed to evaluate the associations between malnutrition and health outcomes.

**Results:**

Data from 334 older adults (mean age: 86.3 ± 4.7 years) were analyzed. Malnutrition was significantly and inversely associated with a history of falls, with non-malnourished individuals being less likely to have experienced a higher number of falls. However, no significant associations were found between malnutrition and the incidence of falls, fall-related fractures, disability (prevalence and incidence), or mortality.

**Conclusion:**

The findings of the present study indicate that malnutrition, as defined by the GLIM criteria, is significantly associated with a history of falls in older adults. Further studies examining important covariates are required to confirm our findings and expand the current knowledge in the field.

## 1 Introduction

Malnutrition (e.g., undernutrition) refers to a health condition characterized by insufficient intake or absorption of nutrients, resulting in significant changes in body composition and a decline in biological function (1). Malnutrition is highly prevalent among older adults and has received considerable attention from international organizations, as its progression is expected to increase the risk of negative health events (2–4).

Nonetheless, these assumptions have been increasingly challenged by empirical findings reporting inconsistent results on this association (5). One possible explanation for these discrepancies is the variation in assessment tools used to evaluate malnutrition. To minimize inconsistencies in study findings, the Global Leadership Initiative on Malnutrition (GLIM) (1) convened leading clinical nutrition societies to establish a standardized method for defining and assessing malnutrition. Subsequently, numerous studies have examined whether malnutrition, as defined by this framework, is significantly associated with health parameters (6). However, data regarding many important outcomes remain limited.

A recent scoping review found that only three studies have investigated the occurrence of falls and fractures as a result of malnutrition, assessed according to the GLIM framework, whereas no investigations have linked this operationalization method with the incidence of disability (6). Furthermore, the majority of studies have been conducted in acute care and hospital settings (6), while community-dwelling older adults, a population at high risk for malnutrition, remain substantially underrepresented in research.

Given the increasing clinical interest in the GLIM criteria and their potential broad applicability, there is a critical need for studies that investigate their predictive value for common adverse outcomes among community-dwelling older individuals. Robust evidence from such studies is essential to strengthen the clinical relevance and utility of the GLIM framework across different care settings.

Thus, the present study was designed to address these knowledge gaps by investigating the associations between malnutrition, operationalized according to the GLIM criteria, and key health-related outcomes—including falls, fall-related fractures, disability, and mortality—particularly among community-dwelling older adults. By focusing on this underexplored population and set of outcomes, our study provides novel insights that contribute to advancing the clinical application of the GLIM criteria in real-world settings.

## 2 Methods

The current investigation utilized data from the Aging and Longevity Study in the Sirente Geographic Area (ilSIRENTE) database (7). This prospective cohort study was carried out in the mountainous community of the Sirente region of Central Italy, located within L’Aquila province in Abruzzo. The population in this region resides in 13 towns and villages, situated at elevations ranging from 800 to 1,400 m above sea level and surrounded by mountainous terrain. While predominantly rural, the area relies primarily on agriculture as its main economic activity. The ilSIRENTE study was developed collaboratively by the Department of Geriatrics at the Università Cattolica del Sacro Cuore (Rome, Italy) and the teaching nursing home Opera Santa Maria della Pace (Fontecchio, L’Aquila, Italy), in partnership with local administrators and primary care physicians from the Sirente Mountain Community Municipalities.

The research adhered to the ethical guidelines outlined in the Declaration of Helsinki and received approval from the Ethics Committee of Università Cattolica del Sacro Cuore. Written informed consent was obtained from all participants or their legal representatives, when necessary, prior to enrollment.

### 2.1 Participants

In October 2003, a comprehensive list of residents in the Sirente area was obtained from the registry offices of all municipalities involved in the study. Registration in these municipal records is mandatory at birth or upon relocation, as it is required to access primary healthcare services. This requirement ensured complete population coverage, including individuals residing in nursing homes.

From this list, potential study participants were identified by selecting all individuals living in the Sirente area born before 1 January 1924. Among the 514 subjects initially screened, 32 men and 53 women had either died or moved away prior to baseline assessment. General practitioners presented the ilSIRENTE study protocol to their eligible patients and invited them to participate. Individuals who initially declined were contacted at least two additional times by the study personnel before being definitively classified as refusals.

Of the 429 eligible individuals, the refusal rate was low (16%), with no significant differences between age and gender. As a result, a total of 364 participants aged 80 years and older were enrolled in the ilSIRENTE study. For the present analysis, only data from participants with complete information required to construct the GLIM criteria and assessment of negative outcomes were included, resulting in a final analytical sample of 334 individuals.

### 2.2 Data collection

Baseline evaluations started in December 2003 and were completed in September 2004. Clinical interviews and functional assessments were carried out at designated study clinics in each town. For participants unable to visit the clinics due to physical or cognitive impairments or transportation challenges, assessments were conducted in their homes. Data on medical history, medications, and lifestyle were collected through validated questionnaires (7). All procedures were conducted by a trained team comprising physicians, nurses, physiotherapists, medical residents, and medical students from the Department of Geriatrics at the Università Cattolica del Sacro Cuore, the teaching nursing home Opera Santa Maria della Pace, and local primary care physicians. The ilSIRENTE study database is under the stewardship of the principal investigator (F. L.).

### 2.3 Malnutrition

Malnutrition was operationalized according to the presence of at least one phenotypic and at least one etiologic criterion, as recommended by the GLIM criteria (1). Phenotypic criteria included the following parameters: (a) unintentional weight loss ≥5% in the last 30 days or ≥10% in the last 180 days, (b) low body mass index (BMI, <22 kg/m^2^), and (c) low muscle mass (appendicular skeletal muscle (ASM) < 20 kg for men and <15 kg for women) (8). Etiologic criteria included: (a) reduced food intake (answered “Yes, a little” or “Yes, a lot” to the question “The amount of food you usually eat has decreased over the last year?”) and b) inflammation (C-reactive protein (CRP) values ≥ 9 mg/L) (9).

### 2.4 Outcomes

Fall history, incident falls, and fall-related fractures were assessed using item 5 of section K of the Minimum Data Set for Home Care (MDS-HC) instrument (10). Participants or their proxies were asked to report any fall events they had experienced during the 90 days prior to the baseline or follow-up visit. The capacity to perform basic (ADL) and instrumental activities of daily living (IADL) was assessed using the subscale H of the MDS-HC instrument (10). The capacity to independently perform 10 ADLs (i.e., mobility in bed, transfer, locomotion in and outside home, dressing upper and lower body, eating, toilet use, personal hygiene, and bathing) in the last 3 days was scored as follows: (0) independent, (i) setup help only, (ii) supervision, (iii) limited assistance, (iv) extensive assistance, (v) maximal assistance, and (vi) total dependence. A score of 0–3 was attributed to the participant’s autonomy to perform seven IADLs (i.e., meal preparation, ordinary housework, managing finance, managing medication, phone use, shopping, and transportation) in the last 7 days, as follows: (0) independent, (i) some help, (ii) full help, and (iii) performed by others. Both ADL and IADL were coded onto an 8-category hierarchical scale, with 0 indicating total independence/capacity and 7 corresponding to total dependence/incapacity (11, 12). Incident disability was operationalized as the development of incapacity to independently perform one or more ADLs, including dressing, eating, toilet use, bathing, mobility in bed, locomotion, and transfer at a 2-year follow-up. Survival status was obtained from the participants’ general practitioners and was confirmed through the National Death Registry (7). Time to death was calculated from the date of the baseline visit to the date of death. All events that occurred up to 10 years after enrollment were included in the analysis.

### 2.5 Covariates and adjustment variables

Body height and weight were measured using a stadiometer and an analog medical scale, respectively. BMI was calculated by dividing the body weight (kg) by height squared (m^2^). Calf circumference was measured on the dominant leg by measuring the widest point (cm) between the ankle and knee joints with an anthropometric tape while the participant was seated. Measurements were rounded to the nearest 0.1 cm. ASM was calculated using the formula provided by the COCONUT Study Group (Grupo de Estudos em Composição Corporal e Nutrição) (13). Physical activity levels were determined through self-reported data. Participants reported their physical activity patterns during middle age (40–60 years) and during the year prior to the interview, selecting from the following categories: (a) almost no physical activity; (b) primarily sedentary with brief periods of light walking or activity; (c) low-intensity activities (e.g., walking, dancing, fishing, and hunting) for 2–4 h per week; (d) moderate-intensity activities (e.g., running, uphill walking, swimming, and gymnastics) for 1–2 h per week or low-intensity activities exceeding 4 h per week; (e) moderate-intensity activities for more than 3 h per week; (f) high-intensity activities on most days; or (g) walking more than 5 km per day on at least 5 days per week. Before data collection, the concepts of low, moderate, and high intensity were clarified to participants. Multimorbidity was defined as the coexistence of two or more of the following conditions: obesity, coronary heart disease, cerebrovascular disease, congestive heart failure, peripheral artery disease, hypertension, lung disease (including chronic obstructive pulmonary disease, emphysema, or asthma), osteoarthritis, diabetes, dementia, Parkinson’s disease, renal failure, and cancer. This definition is widely recognized and frequently applied in studies involving older populations (14). Clinical diagnoses were recorded using Section J of the MDS-HC (10) based on self-report, information gathered from primary care physicians, physical examinations, and comprehensive reviews of clinical documentation, including laboratory tests and imaging examinations. Current smoking was classified as the regular use of tobacco at least once per week for the past year. Participants’ self-assessed economic status was grouped into the following categories: (i) uncertain, (ii) good, (iii) sufficient, and (iv) poor. Marital status, level of formal education, and time elapsed since the last hospital admission were evaluated using questions 4 and 6 from section BB and question 4 from section C of the MDS-HC (10).

### 2.6 Statistical analysis

Continuous variables were expressed as mean ± standard deviation (SD), while categorical and ordinal variables were reported as absolute numbers and percentages. An independent *t*-test was used to examine differences in continuous variables according to the presence of malnutrition. Binary regression analyses were conducted to examine the associations between malnutrition and the incidence of falls, fall-related fractures, and ADL and IADL disabilities. Ordinal regression analyses were conducted to test the associations between malnutrition and history of falls, as well as the capacity to perform ADL and IADL tasks. The Kaplan–Meier and Cox proportional hazards analyses were used to identify predictors of survival. The final models were adjusted for age, sex, BMI, physical activity levels during middle age and in the past year, smoking status, formal education, time since last hospital stay, marital status, self-perceived economic status, and multimorbidity. A significance level of 5% (*p* < 0.05) was used for all tests, and all *p*-values were determined using two-tailed tests. All analyses were performed using SPSS software (version 23.0, SPSS Inc., Chicago, IL, United States).

## 3 Results

### 3.1 Participant characteristics

The main characteristics of study participants according to the presence of malnutrition are shown in Table 1. Participants had a mean age of 86.3 (± 4.7) years, and the majority of the participants were women (67.0%). The prevalence of malnutrition was 22.4%. Malnourished participants were older and had lower BMI and ASM than their non-malnourished peers. No other significant differences were observed between the groups.

**Table 1:** Main characteristics of study participants (*n* = 334).

Variables	Malnourished (*n* = 75)	Non-malnourished (*n* = 259)
Age, years	87.2 ± 4.6	85.3 ± 4.8*
Sex, Female, %	28.4	71.6
BMI, kg/m^2^	23.6 ± 4.5	26.2 ± 4.3*
ASM, kg	11.0 ± 3.7	16.2 ± 5.1*
Current smoking status, %	22.5	75.1
Multimorbidity, %	9.6	26.6
Physically active last year, %	9.0	18.1
Physically active during middle age, %	22.0	76.5
Self-rated economic status, %		
(a) Do not know	0.6	1.2
(b) Good	0.3	0.6
(c) Sufficient	0.9	6.4
(d) Bad	19.5	66.8
Marital status, %		
(a) Married	3.6	24.3
(b) Never married	3.0	6.9
(c) Widowed/separated/divorced	46.4	15.9
Formal education, %		
(a) No schooling	0.6	2.4
(b) 8th grade/less	–	0.6
(c) 9–11 grades	21.6	69.5
(d) High school	–	2.1
(e) Technical or trade school	–	2.1
(f) Some college	–	0.6
(g) Bachelor’s degree	0.3	0.3
(h) Graduate degree	–	–
Time since last hospital stay, %		
(a) No hospitalization in the last 180 days	18.3	66.5
(b) Within the last week	0.6	2.4
(c) Within 8–14 days	0.6	0.9
(d) Within 15–30 days	0.3	1.2
(e) More than 30 days ago	2.7	6.6

ASM, appendicular skeletal muscle; BMI, body mass index. *p < 0.05 vs. malnourished.

### 3.2 Associations between malnutrition and the prevalence of disability and history of falls

The results for the association between malnutrition and the prevalence of disability and history of falls are shown in Table 2. In the unadjusted ordinal analysis, malnutrition was significantly and inversely associated with the number of previous falls and the degree of disability. Non-malnourished individuals were less likely to have experienced a higher number of falls and to exhibit greater dependence on ADL and IADL. When the analysis was adjusted for covariates, the results remained significant for history of falls but not for ADL or IADL disability.

**Table 2:** Unadjusted and adjusted ordinal regression for the associations between malnutrition and disability and history of falls.

	Unadjusted			Adjusted		
	β	CI	*p*-value	β	CI	*p*-value
ADL disability	−1.747	(−2.261, −1.233)	0.001	−0.358	(−1.096, 0.380)	0.342
IADL disability	−1.561	(−2.040, −1.082)	0.001	−0.616	(−1.289, 0.057)	0.073
History of falls	−1.130	(−1.789, −0.471)	0.001	−1.084	(−1.883, −0.285)	0.008

ADL, activities of daily living; IADL, instrumental activities of daily living; CI, confidence interval.

### 3.3 Associations between malnutrition and the incidence of disability, falls, and fractures

The results for the associations between malnutrition and the incidence of disability, falls, and fractures are shown in Table 3. In the unadjusted analysis, malnutrition was significantly associated with the occurrence of falls and fall-related fractures. However, the results were no longer significant after adjusting for covariates. No other significant associations were observed with fractures or disability.

**Table 3:** Unadjusted and adjusted binary regression for the associations between malnutrition and the incidence of negative health events.

	Unadjusted			Adjusted		
	OR	CI	*p*-value	OR	CI	*p*-value
Disability	1.566	(0.768, 3.191)	0.217	0.959	(0.411, 2.238)	0.923
Falls	3.399	(1.523, 7.322)	0.003	2.088	(0.847, 5.143)	0.110
Fractures	1.666	(0.424, 6.539)	0.465	0.972	(0.186, 5.078)	0.973
Fall-related fractures	3.339	(1.523, 7.322)	0.003	2.088	(0.847, 5.143)	0.110

CI, confidence interval; OR, odds ratio.

### 3.4 Associations between malnutrition and death

The results for the association between malnutrition and death are reported in Table 4. A significant association between malnutrition and mortality was observed in the unadjusted analysis. However, significance was lost when the model was adjusted for covariates.

**Table 4:** Kaplan–Meier and Cox regression for the predictive capacity of malnutrition toward death.

	Kaplan–Meier		Cox regression	
	HR	*p*-value	HR	*p*-value
Malnutrition	18.109	0.001	1.321	0.075

HR, hazard ratio.

## 4 Discussion

The main findings of this study indicate that malnourished older adults were more likely to have experienced a higher number of falls, even after adjusting for multiple covariates. However, no significant associations were found between malnutrition and the incidence of falls, fractures, or fall-related fractures. In addition, our results indicate that ADL and IADL disability were not crosssectional or longitudinally associated with malnutrition in older adults.

The results of the present study are in line with previous investigations that found significant associations between malnutrition and a history of falls in older adults. Neyens et al. (15) reported that undernourished older adults living in long-term care institutions in the Netherlands were more likely to have experienced falls in the 30 days prior to data collection compared to their non-undernourished peers. Similarly, Meijers et al. (16) found that older adults who had fallen were more often malnourished than non-fallers, even after adjusting for covariates. The importance of examining our study population was further highlighted by Eglseer et al. (17) who observed that malnutrition, assessed using the Malnutrition Universal Screening Tool (MUST) assessment tool, was significantly associated with in-hospital falls among Austrian older adults aged 80 + years, but not in those aged 65–79 years.

A possible explanation for these associations is the detrimental impact of malnutrition on the neuromuscular system. Several diagnostic criteria under the GLIM criteria (i.e., weight loss, low BMI, and reduced muscle mass) are associated to muscle atrophy, impaired force production, and mobility limitations (18–20). Furthermore, there is an overlap between the phenotypic criteria of the GLIM framework and geriatric conditions such as sarcopenia and frailty (8, 21). Consequently, undernourished older adults may exhibit significant neuromuscular impairments (3), which could increase their susceptibility to falls (2, 22).

However, the cross-sectional associations between malnutrition and falls were not confirmed in the longitudinal analysis. Moreover, the associations with fall-related fractures were no longer significant, after adjusting for covariates, despite being significant in the unadjusted analysis. There are conflicting results regarding the ability of malnutrition to predict falls and fractures in older adults, with investigations reporting significant (23) or null associations (4, 24). These results likely reflect the multifactorial nature of falls, which may play a more determinant in examining their incidence instead of previous events.

Falls commonly occur as a combination of intrinsic (e.g., mobility problems, balance, and visual problems) and extrinsic (floor conditions, trip, and slip) factors (24, 25). In the present study, we controlled the analysis for many potential intrinsic factors, including age, BMI, physical activity levels during middle age and in the last year, and multimorbidity (24, 25). However, the lack of adjustment for extrinsic factors hampers a thorough interpretation of our results, suggesting that the examination of both intrinsic and extrinsic factors is required to effectively explore the association between malnutrition and falls.

Another important aspect is that malnutrition and covariates were examined only at baseline, without accounting for their trajectories over the follow-up period. This limitation is noteworthy, as numerous studies have demonstrated that changes in lifestyle habits are significantly associated with health events (26, 27). This scenario may also explain the lack of association observed between malnutrition and the capacity to perform ADL and IADL. Sanchez-Sanchez et al. (28), for example, revealed that older adults who increased their physical activity levels during follow-up had a lower risk of disability. Regarding the underlying conditions linking malnutrition and disability, Álvarez-Bustos et al. (26) reported that older adults who experienced a worsening of frailty were at a higher risk of disability compared to those who did not develop frailty and those who remained at the same degree of frailty severity.

A complementary perspective to these premises is that a majority of the studies that reported significant associations between malnutrition and disability focused on older adults in specific conditions, such as home care (27), institutionalization (29), patients with cancer (30), and heart failure (31). Capturing the trajectories from the community to other specific health conditions is challenging and requires more detailed, condition-specific investigations.

In the present study, malnutrition did not increase the risk of death in octogenarians. Only two other studies have investigated this association using the GLIM criteria for malnutrition. Yeung et al. (3) observed relatively healthy, community-dwelling Chinese older adults recruited from community centers over 14 years and found that those who were undernourished had a higher risk of death. Similar findings were reported by Sanchez-Rodriguez et al. (32), who analyzed data from the SarcoPhAge study, involving older adults recruited from an outpatient clinic and followed for up to 4 years. A possible explanation for these divergent results may be based on the covariates examined. Indeed, Yeung et al. (3) adjusted their analysis for living status (e.g., living alone or with a partner) and the community status ladder. Social isolation, loneliness, and living alone have been significantly associated with increased mortality, which potentially mediated by higher engagement in risky health behaviors and reduced seeking of medical treatment (33). Although participants of the IlSIRENTE study were recruited from small mountainous communities, where people are highly likely to be socially connected, this aspect warrants further investigation. Regarding Sanchez-Rodriguez et al. (32), the authors employed a more diverse statistical approach than that used in the present study, analyzing a different set of covariates. In particular, the authors did not examine important parameters independently associated with mortality, such as BMI (34), physical activity levels (35), and previous hospitalization (36).

The findings of the present study expand the current knowledge by indicating that malnutrition, as defined by the GLIM criteria, is also associated with a history of falls in community-dwelling older adults. These findings need further confirmation and a more detailed exploration of the components of the GLIM criteria, mainly regarding the inflammation criteria. Although CRP is endorsed by the GLIM criteria as a valuable supportive proxy measure (1) due to its sensitivity in reflecting variations in the inflammatory state (37), examining the burden of diseases associated with inflammation (e.g., heart failure), with or without additional inflammatory markers (e.g., interleukins), as part of the etiological criteria, could provide further insights.

A more detailed examination of the GLIM criteria could be particularly valuable in low-income countries, where malnutrition is highly prevalent (38). Despite the widespread challenge of malnutrition in these regions, studies focusing on the application of the GLIM criteria in such populations are still scarce and deserve further exploration. Understanding how the GLIM criteria perform in the context of resource-limited settings could provide critical insights into the nutritional status of vulnerable populations and inform public health strategies aimed at addressing malnutrition more effectively.

The present study is not without limitations. First, longer follow-up periods may be necessary to identify associations between malnutrition and mortality. For example, Yeung et al. (3) utilized a 14-year follow-up period. Second, we compared our study results with investigations using other malnutrition assessment tools, due to the limited number of studies using the GLIM criteria. It is important to note that various malnutrition assessment tools capture different health parameters and are only barely/moderately correlated (27, 32). Third, malnutrition in this study was assessed using a modified version of the GLIM criteria. Fourth, covariates, including physical activity levels, were assessed using self-reported measures. Fifth, important dietary aspects that might influence the associations examined in the present study, such as calorie and protein intake, were not recorded. Sixth, malnutrition may influence bone metabolism, potentially increasing the risk of osteoporosis, which, in turn, can contribute to a higher incidence of falls and fractures. This relationship represents a significant determinant of both falls and fractures, highlighting the need for further exploration in future research. Understanding the intersection between malnutrition and bone health could provide valuable insights into targeted interventions to reduce fall-related injuries in older adults. Finally, the ilSIRENTE study was designed to examine the entire population of octogenarians from a specific Italian region with very similar lifestyle characteristics. As a consequence, our study examined a cohort of relatively healthy, very old adults living in a mountainous region, and caution should be warranted when generalizing these findings to other populations.

In conclusion, the findings of the present study suggest that malnutrition, as defined by the GLIM criteria, is significantly associated with a history of falls in older adults. More studies examining important covariates are required to confirm our findings and expand the current knowledge, primarily regarding other key health events.

## Data Availability

The raw data supporting the conclusions of this article will be made available by the authors, without undue reservation.
